# Smartphone measures of day-to-day behavior changes in children with autism

**DOI:** 10.1038/s41746-018-0043-3

**Published:** 2018-08-14

**Authors:** Rebecca M. Jones, Thaddeus Tarpey, Amarelle Hamo, Caroline Carberry, Catherine Lord

**Affiliations:** 1 The Center for Autism and the Developing Brain, Weill Cornell Medicine, 21 Bloomingdale Road, White Plains, NY 10605 USA; 2 The Sackler Institute for Developmental Psychobiology, Weill Cornell Medicine, 1300 York Avenue, New York, NY 10065 USA; 3 Division of Biostatistics, Department of Population Health, New York University, New York, NY 10016 USA

**Keywords:** Outcomes research, Paediatric research

## Abstract

Smartphones offer a flexible tool to collect data about mental health, but less is known about their effectiveness as a method to assess variability in children’s problem behaviors. Caregivers of children with autism completed daily questions about irritability, anxiety and mood delivered via smartphones across 8-weeks. Smartphone questions were consistent with subscales on standard caregiver questionnaires. Data collection from 7 to 10 days at the beginning and 7 to 10 days at the end of the study were sufficient to capture similar amounts of variance as daily data across 8-weeks. Other significant findings included effects of caregiver socioeconomic status and placebo-like effects from participation even though the study included no specific treatment. Nevertheless, single questions via smartphones collected over relatively brief periods reliably represent subdomains in standardized behavioral questionnaires, thereby decreasing burden on caregivers.

## Introduction

There is increasing demand for quick, real-time caregiver and self reports on smartphones to replace longer symptom assessment questionnaires as outcome measures of environments beyond clinics in psychiatry and pediatric medicine.^1,2^ In developmental populations, smartphones have the potential to capture variability in children’s mood or challenging behaviors but many unknowns exist about implementation outside the clinic.

The goal of the present study was to identify effective methods to scale smartphones in the home to collect data from caregivers about children’s behavior. Daily smartphone delivery of questions about problem behaviors to caregivers of children with autism was tested across 8-weeks. No specific intervention was offered; therefore, significant changes in children’s symptoms over time were not predicted; rather we expected fluctuations commonly associated with autism.

## Results

Smartphone questions addressing irritability, mood, disruptive behavior, and anxiety were compared to similar subscales on standard caregiver questionnaires (Table 1; Supplementary Table 1). Over 8-weeks, caregivers reported improvement in their child’s mood, irritability and disruptive behaviors during treatment as usual.^3^ More educated caregivers rated their children’s irritability, disruptive behavior and anxiety, but not mood, more severely than less educated caregivers.

**Table 1:** Summary of comparison between questions on the smartphone and standardized questionnaires

Smartphone vs. Standard Questionnaire	Main Effect (|z|-statistic)	Day (|z|-statistic)	Correlation with TV |r|-value	Caregiver Education (|z|-statistic)
Irritable vs. ABC irritability	2.438*	3.384***	0.442	2.658**
Irritable vs ABC hyperactivity	1.759	3.385***	0.499*	2.728*
Irritable vs VAS disruptive	3.511***	3.336***	0.680***	2.744**
Disruptive vs ABC irritability	2.667**	2.741**	0.364	3.445***
Disruptive vs ABC hyperactivity	2.599**	2.728**	0.39	3.428***
Disruptive vs VAS disruptive	4.180***	2.697**	0.642**	3.649***
Anxiety vs CBCL internalizing	1.925	2.133*	0.185	2.294*
Anxiety vs VAS anxiety	3.131**	1.907	0.268	3.107**

Day was included as a covariate. Correlation with Total Variance (TV) demonstrates relationship between TV and standard questionnaires

*CBCL* Child Behavior Checklist, *ABC* Aberrant Behavior Checklist, *VAS* Visual Analogue Scale

*p < 0.05, **p < 0.01, ***p < 0.001. Main effects |z|’s>2.43 remain significant at alpha=0.05 using Hommel’s multiplicity adjustment to control the familywise error

**Fig. 1:**
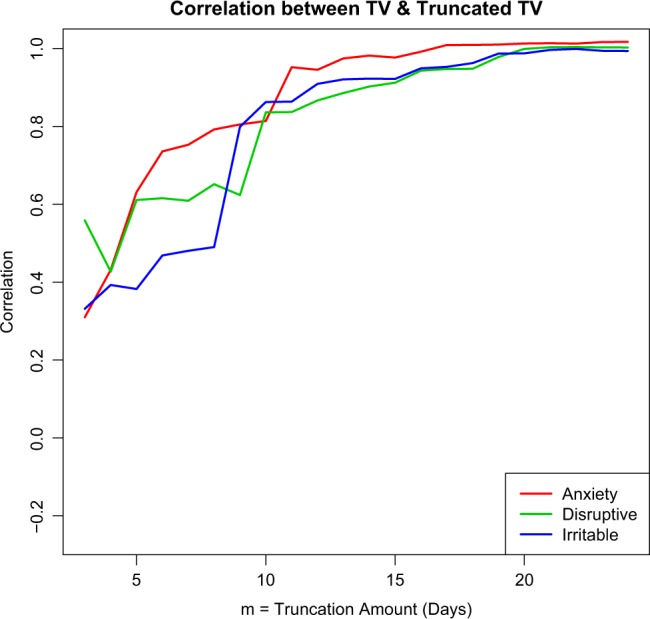
Plot of correlation (r) between Total Variance (TV) and the Truncated TV for the range of truncation values m = 3; 4; 5;…24. Each of the curves corresponds to one of the smartphone questions. As m gets larger, the truncated sample gets closer to the entire sample and hence, the r-values approach 1. After about a truncation of m = 10 days at the beginning and end of the study, the correlation between TV and the truncated TV is significant for all outcomes (and is significant for outcomes by about 7 days)

Higher total variance (TV), an overall measure of variability, on smartphone questions that targeted mood, irritability and disruptive behavior corresponded to more severity on standard paper questionnaires. Increased variance on one smartphone question was associated with increased variance on another (Supplementary Figure 1). As illustrated in Fig. 1, 7–10 days at the beginning and 7–10 days at the end of the study (truncated total variance) were sufficient to capture similar amounts of variance as 8-weeks of daily data (total variance).

## Discussion

Caregivers assessed their child’s behavior at home via questions on a smartphone. Single smartphone questions captured day-to-day variation in mood, anxiety and irritable/disruptive behaviors in children with autism, as indicated by significant relationships between smartphone questions and standardized questionnaires.

Reports from 7 to 10 days at the beginning and end of the study were equally valuable as 8-weeks of daily data. More educated caregivers rated their child’s behaviors more severely, indicating the importance of controlling for socioeconomic background. In addition, as occurred with the questionnaires, caregivers reported symptoms improving over time without explicit treatment.^3^ Frequent reminders from study staff to increase caregiver participation were necessary. Replication findings in a more diverse sample will be important. Nevertheless, single questions on smartphones over 7 to 10 days at the beginning and end of a study offer the potential to represent irritability, mood, anxiety and disruptive behavior in children as effectively as questionnaires or daily data collection over 8-weeks.

## Method

In total 20, 5–13 year-old participants with autism and their families were recruited through the Center for Autism and the Developing Brain (CADB) in White Plains, NY. Caregivers gave written consent; when possible, children 7 years and above assented. Weill Cornell Medicine’s IRB approved the study (#1405015095).

Participants and their caregivers completed an 8-week study consisting of home use of smartphones and clinic visits in weeks 1,4 and 8.^3^ In total 14 mothers, 3 fathers, 2 mothers & fathers, and 1 other family member completed the study. 55% had graduate degrees, 30% bachelor’s degrees, 5% some college and 10% high school diplomas.

Caregivers completed the Aberrant Behavior Checklist (ABC),^4^ Child Behavior Checklist (CBCL),^5^ Positive Affective and Negative Affective Scale (PANAS),^6^ Visual Analogue Scale (VAS) for anxiety and disruptive behavior during week 1 (T1) and week 8 (T2).

A designated caregiver installed the Janssen Autism Knowledge Engine (JAKE™) application during the first clinic visit.^7^ During weeks 1, 4 and 8, caregivers completed smartphone questions every day; during remaining weeks they answered questions at least 3 times per week. Questions addressed the child being tense/worried, irritable, and disruptive on an 8-point scale (0-very to 7-not at all) at the moment.

### Data analysis

The internalizing subscale of the CBCL, the hyperactivity and irritability subscales of the ABC, the positive and negative mood scales of the PANAS and VAS for anxiety and disruptive behaviors were collected at T1 as representative of well documented standardized questionnnaires measuring roughly equivalent concepts. There were 456 responses on average per smartphone question. Mixed-effect ordinal logistic regression models assessed the consistency between caregiver report on the smartphone and questionnaires, with a subject-level random effect. A separate analysis included Day (1-up to 65), with a second analysis including Caregiver Education in the regression models. The clmm function from the ordinal package in R was used to fit the models (see Table 1 and Supplementary Table 1 for a summary of the regression models).

Total variance (TV) was computed for ordinal values for each smartphone question for each participant. TV was then calculated with only the first and last *m* days of data for a range of values of *m*. The correlation between TV and TV with data truncated at *m* day was computed for the range of truncation values *m* (Fig. 1). Pearson correlations, r, were computed between TV on the smartphone and paper questionnaires (Table 1).

### Data availability

We will share the data upon reasonable request.

## Electronic supplementary material

Supplementary Material

## Electronic supplementary material

**Supplementary information** accompanies the paper on the *npj Digital Medicine* website (10.1038/s41746-018-0043-3).
