# Supplementary material for: Smartphone measures of day-to-day behavior changes in children with autism
Source: NPJ Digit Med. 2018 Aug 14;1:34. doi: 10.1038/s41746-018-0043-3 (PMC6550261; doi:10.1038/s41746-018-0043-3)
Supplement: Supplementary file 1 — Supplementary Material [file 41746_2018_43_MOESM1_ESM.pdf]

Supplementary Material

Supplementary Methods

A diagnosis of ASD in the child was confirmed prior to participation by a licensed clinician at CADB using the Autism Diagnostic Observation Schedule (ADOS)[8] or the Adapted ADOS as well as the Autism Diagnostic Interview- Revised (ADI-R)[9]. Calibrated Severity Scores (CSS) for Social Affect (SA) and Restricted and Repetitive Behaviors (RRB) were calculated from the Autism Diagnostic Observation Schedule (ADOS)[8]. IQ scores were calculated from developmentally appropriate cognitive testing. In addition to the questionnaires outlined in the manuscript, caregivers also completed the Caregiver Strain Questionnaire (CSQ)[10]

JAKE™ was modified for the purpose of this study with permission. The caregiver received a daily reminder at 8:00pm to use JAKE™. If the caregiver was not using the application, an examiner reached out to request his or her continued participation. Caregivers also received smartphone questions addressing irritability, negative mood, disruptive behavior, and anxiety on a 4-point scale. Of note, there was no mood question on the 8-point scale, but otherwise there was no difference in the content of the 8-point versus 4-point scale questions.

In separate regression models with each smartphone question and standardized questionnaire, we tested the extent child characteristics (verbal IQ, age and autism severity, as measured by the ADOS CSS SA and CSS RRB) influenced how caregivers reported about their child on the smartphone. Separate regression models were used to test whether caregiver education, including caregiver stress reported on the CSQ, influenced reporting on the smartphone.

Supplementary Results

The results from the 4-point scale are summarized in Supplementary Table 1. Of note, only the question about mood demonstrated a change across time in how caregivers perceived their child's behavior. There was no relationship between caregiver education and the questions on the 4-point scale. There was no influence of the child's age or autism severity on caregiver reporting (all *p*'s > 0.05). Child's higher VIQ was associated with more positive mood ratings($z=2.182$; $p < 0.05$). There was no influence of caregiver stress (CSQ) on caregiver reporting on either the 4-point or 8-point scale questions.

The changes across days that we observed in the 8-point scales were generated by analyzing data across all days of the study, whereas our prior work analyzed only week 1 and week 8 data and the previous work found a trend in improvement in caregiver reporting on the smartphone[3].

**Smartphone vs. Standard Questionnaire**	**Main Effect (|z|-statistic)**	**Day (|z|-statistic)**	**Correlation with TV |r|-value**
4-point mood vs PANAS Pos	0.826	2.935**	0.201
4-point mood vs PANAS Neg	4.0495***	2.863**	0.625**
4-point disruptive vs ABC irritability	2.684**	0.259	0.412
4-point disruptive vs ABC hyperactivity	2.762**	0.315	0.569**
4-point disruptive vs VAS disruptive	3.672***	0.288	0.423

4-point irritable vs ABC irritability	3.327***	1.214	0.165
4-point irritable vs ABC hyperactivity	2.357*	1.302	0.274
4-point irritable vs VAS disruptive	5.478***	1.249	0.225
4-point anxiety vs CBCL internalizing	3.223**	1.219	0.295
4-point anxiety vs VAS anxiety	3.251**	1.112	0.255

Supplementary Table 1: Summary of comparison between 4-point scale questions on the smartphone and standardized questionnaires. Day was included as a covariate. Correlation with Total Variance (TV) demonstrates relationship between TV with the standard questionnaire. PANAS=Positive and Negative Affect Schedule; CBCL=Child Behavior Checklist; ABC=Aberrant Behavior Checklist; VAS=Visual Analogue Scale. * $p < 0.05$, ** $p < 0.01$, *** $p < 0.001$. Using Hommel's multiplicity adjustment to control the familywise error rate at alpha=0.05, |z|'s > 2.67 correspond to significant effects.

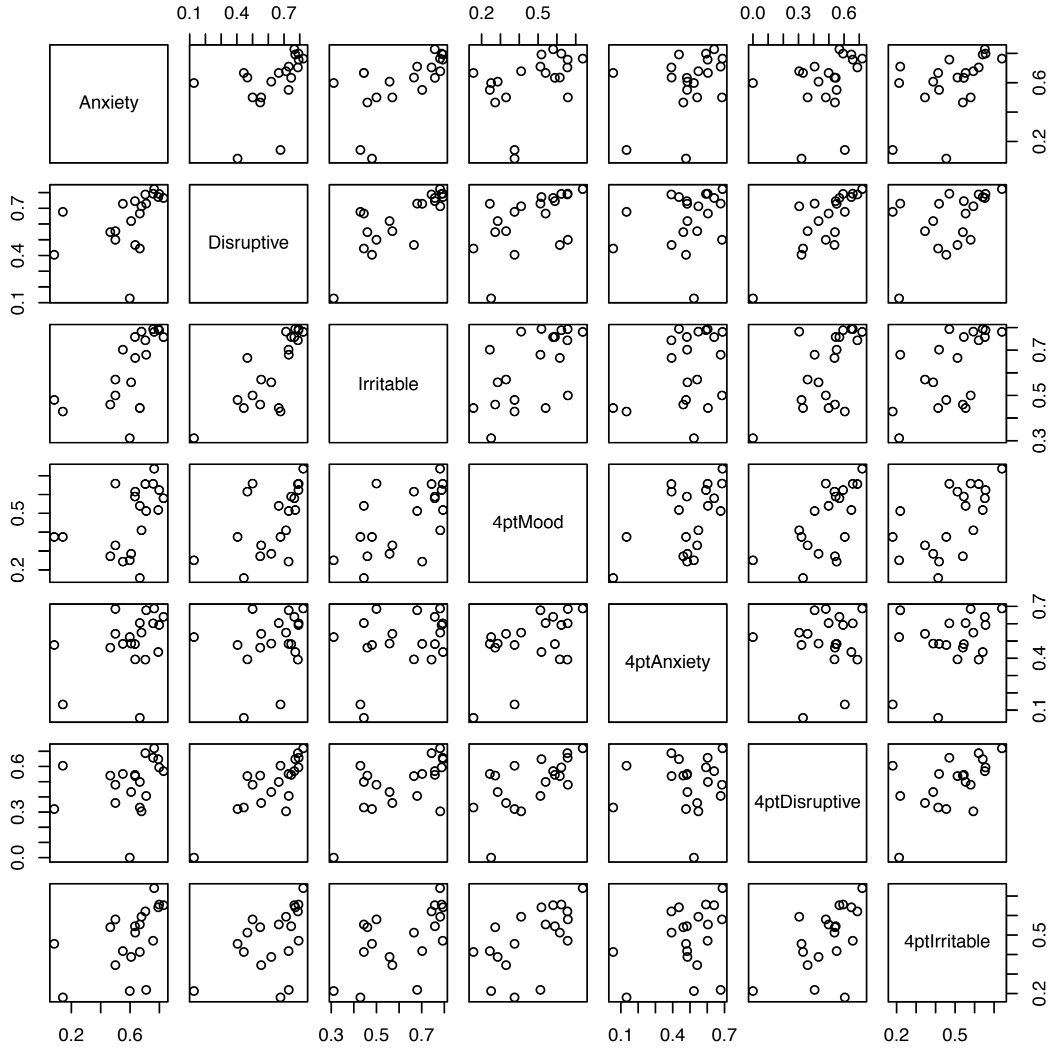

Supplementary Figure 1

Plots of pairwise estimates of the total variance for each of the smartphone questions.

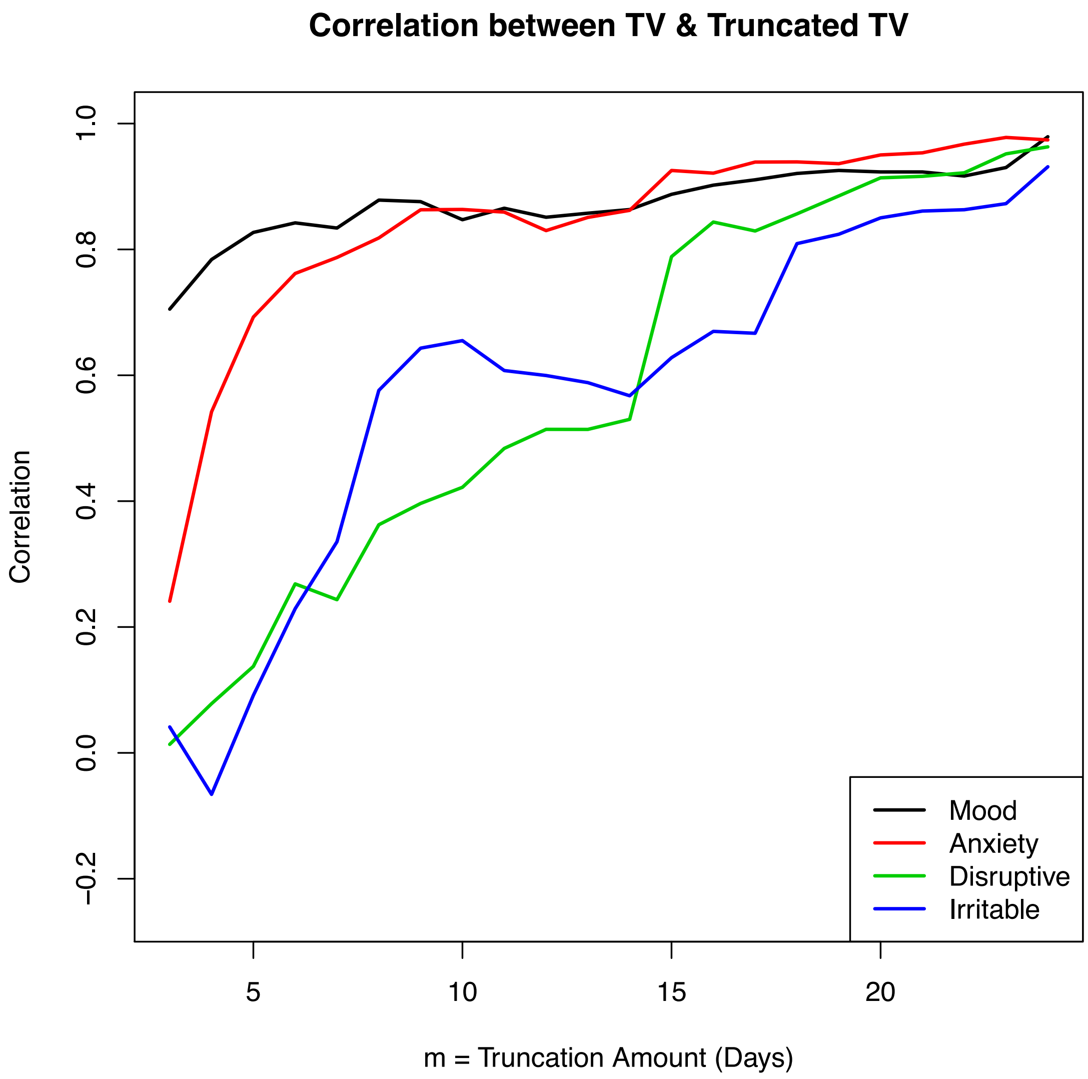

Correlation between TV & Truncated TV
Correlation
m = Truncation Amount (Days)
1.0
0.8
0.6
0.4
0.2
0.0
−0.2
5
10
15
20
Mood
Anxiety
Disruptive
Irritable

Supplementary Figure 2: Plot of correlation (r) between Total Variance (TV) and the Truncated TV for the range of truncation values m = 3; 4; 5…24. Each of the curves corresponds to one of the 4-point scale smartphone questions. As m gets larger, the truncated sample gets closer to the entire sample and hence, the r-values approach 1. After about a truncation of m = 10 days at the beginning and end of the study, the correlation between TV and the truncated TV is significant for all outcomes (and is significant for most outcomes by about 7 days).
